# Towards sustainable partnerships in global health: the case of the CRONICAS Centre of Excellence in Chronic Diseases in Peru

**DOI:** 10.1186/s12992-016-0170-z

**Published:** 2016-06-02

**Authors:** J. Jaime Miranda, Antonio Bernabé-Ortiz, Francisco Diez-Canseco, Germán Málaga, María K. Cárdenas, Rodrigo M. Carrillo-Larco, María Lazo-Porras, Miguel Moscoso-Porras, M. Amalia Pesantes, Vilarmina Ponce, Ricardo Araya, David Beran, Peter Busse, Oscar Boggio, William Checkley, Patricia J. García, Luis Huicho, Fabiola León-Velarde, Andrés G. Lescano, David C. Mohr, William Pan, David Peiris, Pablo Perel, Cristina Rabadán-Diehl, Maria Rivera-Chira, Katherine Sacksteder, Liam Smeeth, Antonio J. Trujillo, Jonathan C. K. Wells, Lijing L. Yan, Héctor H. García, Robert H. Gilman

**Affiliations:** CRONICAS Centre of Excellence in Chronic Diseases, Universidad Peruana Cayetano Heredia, Av. Armendáriz 497, Miraflores, Lima 18, Peru; School of Medicine, Universidad Peruana Cayetano Heredia, Lima, Peru; School of Public Health and Administration, Universidad Peruana Cayetano Heredia, Lima, Peru; Division of Internal Medicine, Hospital Nacional Cayetano Heredia, Lima, Peru; Faculty of Epidemiology and Population Health, London School of Hygiene and Tropical Medicine, London, UK; Division of Tropical and Humanitarian Medicine, Geneva University Hospitals, Geneva, Switzerland; University of Geneva, Geneva, Switzerland; Instituto de Estudios Peruanos, Lima, Peru; Division of Non-Communicable Diseases, Dirección General de Salud de las Personas, Ministerio de Salud, Lima, Peru; Division of Pulmonary and Critical Care, School of Medicine, Johns Hopkins University, Baltimore, MD USA; Department of International Health, Johns Hopkins Bloomberg School of Public Health, Baltimore, MD USA; Department of Pediatrics, Instituto Nacional de Salud del Niño, Lima, Peru; School of Medicine, Universidad Nacional Mayor de San Marcos, Lima, Peru; School of Sciences, Universidad Peruana Cayetano Heredia, Lima, Peru; Department of Parasitology, and Public Health Training Program, USA Naval Medical Research Unit No. 6 (NAMRU-6), Lima, Peru; Center for Behavioral Intervention Technologies, Feinberg School of Medicine, Northwestern University, Chicago, IL USA; Division of Environmental Science and Policy, Nicholas School of the Environment, Duke University, Durham, NC USA; The George Institute for Global Health, University of Sydney, Sydney, New South Wales Australia; World Heart Federation, Geneva, Switzerland; Office of Global Affairs, U.S. Department of Health & Human Services, Washington, DC USA; Childhood Nutrition Research Centre, UCL Institute of Child Health, University College London, London, UK; Global Health Research Center, Duke Kunshan University, Kunshan, Jiangsu China; Center for Global Health – Tumbes, Universidad Peruana Cayetano Heredia, Tumbes, Peru; Cysticercosis Unit, Instituto Nacional de Ciencias Neurológicas, Lima, Peru; Asociación Benéfica PRISMA, Lima, Peru

**Keywords:** Partnerships, Low- and middle-income countries, Team management, Capacity building, Training

## Abstract

Human capital requires opportunities to develop and capacity to overcome challenges, together with an enabling environment that fosters critical and disruptive innovation. Exploring such features is necessary to establish the foundation of solid long-term partnerships. In this paper we describe the experience of the CRONICAS Centre of Excellence in Chronic Diseases, based at *Universidad Peruana Cayetano Heredia* in Lima, Peru, as a case study for fostering meaningful and sustainable partnerships for international collaborative research. The CRONICAS Centre of Excellence in Chronic Diseases was established in 2009 with the following Mission: “We support the development of young researchers and collaboration with national and international institutions. Our motivation is to improve population’s health through high quality research.” The Centre’s identity is embedded in its core values — generosity, innovation, integrity, and quality— and its trajectory is a result of various interactions between multiple individuals, collaborators, teams, and institutions, which together with the challenges confronted, enables us to make an objective assessment of the partnership we would like to pursue, nurture and support. We do not intend to provide a single example of a successful partnership, but in contrast, to highlight what can be translated into opportunities to be faced by research groups based in low- and middle-income countries, and how these encounters can provide a strong platform for fruitful and sustainable partnerships. In defiant contexts, partnerships require to be nurtured and sustained. Acknowledging that all partnerships are not and should not be the same, we also need to learn from the evolution of such relationships, its key successes, hurdles and failures to contribute to the promotion of a culture of global solidarity where mutual goals, mutual gains, as well as mutual responsibilities are the norm. In so doing, we will all contribute to instil a new culture where expectations, roles and interactions among individuals and their teams are horizontal, the true nature of partnerships.

## Abstract (Spanish)

Además de la necesidad de contar con un ambiente proactivo que impulse la innovación crítica y disruptiva, el capital humano requiere tanto de oportunidades para su desarrollo como de capacidades para asumir y sobreponerse a retos. Explorar dichas características permitirá establecer las bases para asociaciones (en Inglés *partnerships*) sólidas y duraderas. En este artículo se describe la experiencia de CRONICAS Centro de Excelencia en Enfermedades Crónicas de la Universidad Peruana Cayetano Heredia en Lima, Perú, como un caso de promoción de asociaciones valiosas y sostenibles para el desarrollo de investigaciones colaborativas a nivel internacional. La Misión de CRONICAS Centro de Excelencia en Enfermedades Crónicas, establecido en el año 2009, indica que “Apostamos por la formación de jóvenes investigadores y la colaboración con instituciones locales e internacionales. Nuestra motivación es mejorar la salud de la población a través de investigación de alta calidad”. La identidad del Centro se refleja en sus valores, —generosidad, innovación, integridad y calidad— y la trayectoria del mismo es el reflejo de múltiples interacciones entre individuos, colaboradores, equipos e instituciones. Sumadas a los retos afrontados, este recorrido se traduce en experiencias concretas que alimentan una evaluación objetiva apuntando a definir un perfil o tipo de asociaciones que ameritan alimentarse y protegerse. No se pretende brindar ejemplos de un perfil particular de asociación exitosa, sino, al contrario, resaltar y transmitir potenciales oportunidades para otros grupos de investigación también ubicados en países de ingresos bajos y medios, y cómo dichas experiencias pueden proveer de una amplia plataforma que permita la generación de asociaciones fructíferas y sostenibles. En contextos con limitaciones y retos cotidianos, las asociaciones requieren ser amamantadas, cuidadas y sostenidas. El reconocer que las distintas asociaciones no son ni deberían ser idénticas entre ellas nos permite aprender de la evolución de las mismas, de sus éxitos y sus obstáculos, así como de sus fallas. Esto se traduciría ulteriormente en la promoción de una cultura de solidaridad global en donde los objetivos comunes junto con los beneficios comunes, de la mano de las responsabilidades comunes, sean la norma. De esta manera, todas las partes y actores involucrados contribuyen a instilar una nueva cultura de horizontalidad a nivel de expectativas, roles e interacciones entre individuos y sus equipos, es decir la verdadera naturaleza de un *partnership*.

## Background

The global challenge of non-communicable diseases requires local and international actors working together and effectively to accomplish major achievements, likely of mutual benefit, for example poverty reduction [1, 2]. The World Health Organization’s Global Action Plan [3] states the importance of promoting and supporting national capacity for high-quality research and for developing prevention and control strategies.

The CRONICAS Centre of Excellence in Chronic Diseases, established at *Universidad Peruana Cayetano Heredia* in Peru, has achieved a substantial track record in securing internationally competitive research grant support [4], being a key actor in major awarded projects totalling approximately ~ USD $17.5 million dollars. Whilst research funding can be a powerful indicator, it is an incomplete marker of capacity building. It conceals significant investments and achievements in terms of another major success factor: trained, engaged and committed human capital and talent. The CRONICAS Centre of Excellence in Chronic Diseases was established in 2009 with the following Mission: “We support the development of young researchers and collaboration with national and international institutions. Our motivation is to improve population’s health through high quality research.” The Centre’s identity is embedded in its core values — generosity, innovation, integrity, and quality— and a list of ongoing projects is available at http://en.cronicas-upch.pe/proyectos/.

Research does not happen in isolation. For talent to strive, it is important to leverage existing research platforms and institutional support [4, 5]. Furthermore, research needs partnerships beyond specific scientific topics or projects, partnerships that foster and develop capacity building. As with any organisation, human capital requires opportunities to develop and capacity to overcome challenges. It also needs an enabling environment fostering critical and disruptive innovation [6]; one where working in partnerships is embedded in day-to-day interactions. In this regard, much of the success of the CRONICAS Centre of Excellence is largely due to the nurturing environment or ecosystem of its host institution, the *Universidad Peruana Cayetano Heredia*, one that is largely prone to support scientific research [7–10] alongside with global health training and collaboration [11, 12]. This environment largely explains the leading track record of this institution in promoting science within Peru and the Latin American region [13]. In addition to this, the establishment of the CRONICAS Centre of Excellence in Chronic Diseases in Peru benefited from having a platform to leverage collaborations with other Centres of Excellence with different degrees of development and expertise, thus leapfrogging the chances to maximise opportunities for collaborative work [5, 14].

Partnership, especially partnerships in health, have been previously explored in terms of its ‘success’, showing that such conceptualization yields a strong predominance of evaluations of processes rather than outcomes of success per se [15]. Another entry point for the evaluation of partnerships has been through a framework that operationalizes partnership synergies [16]. In this paper we describe the experience of the CRONICAS Centre of Excellence as a case study for fostering meaningful and sustainable partnerships for international collaborative research under the umbrella of non-communicable diseases. The Centre’s trajectory is a result of various interactions between multiple individuals, collaborators, teams, and institutions, which together with the challenges encountered, enables us to make an objective assessment of the partnership we would like to pursue, nurture and support.

We do not intend to provide a single example of a successful partnership, but in contrast, to highlight the many challenges and opportunities faced by research groups based in low- and middle-income countries, and how these encounters can provide a strong platform for fruitful and sustainable partnerships. Our Centre operates in a country that has recently been categorised as an upper-middle-income economy. Peru, however, with over a quarter of its population living on poverty [17], lacks behind in many major social indicators. For example, it has one of the lowest per capita health expenditures in the region [18, 19] and one of the lowest scores in quality of education [20]. This is a particularly defiant context, in great need for partnerships to be established, nurtured and sustained in order to secure a transition “from construction workers to architects” [21]. Acknowledging that all partnerships are not and should not be the same, we also need to learn from the evolution of such relationships, its key successes, hurdles and failures.

## Review

### Principles for Partnerships

Research projects are time-bound, having an end-date, and usually prioritize research objectives over the development of young researchers. Funding cycles and priorities are constantly changing, overarching inter-institutional agreements focus largely on institutions and less so on people, and funding availability for scientific research remains a constant concern. These factors are beyond the direct influence of many research teams, who need to find ways of managing such uncertain scenario.

From a pragmatic point of view, we contend that partnerships are key to protect and nurture our human capital, and to enhance our local talent [14, 21–25]. Yet, we are also aware that predominant approaches of partnerships, such as North-led, high-income country led, medical professional led, and its combinations, have to be constantly revisited.

Partnerships require several forms and degrees of interaction, communication, and mutuality, as stated by the Swiss Commission for Research Partnerships with Developing Countries (KFPE) [26, 27]. This approach has challenged our team to defend horizontality, mutuality, and willingness to accommodate to the interests of the majority, and to live by our Centre’s values of generosity, innovation, integrity and quality.

The eleven principles recommended by the KFPE guide are 1) Set the agenda together, 2) Interact with stakeholders, 3) Clarify responsibilities, 4) Account to beneficiaries, 5) Promote mutual learning, 6) Enhance capacity, 7) Share data and networks, 8) Disseminate results, 9) Pool profits and merits, 10) Apply results, and 11) Secure outcomes. Some items from this list, specifically principles 1, 3, 5, 6, 7, and 9, call for a strong organizational culture, a culture where shared values between local and international partners should reign. The remaining set of principles also calls for a proactive responsibility with external actors.

Throughout this manuscript, we describe why and how such coherence is needed to sustain real long-term partnerships, such as the ones nurtured by CRONICAS and its international collaborators. Addressing partnerships in isolation would be irresponsible, and more concretely, we cannot address partnership without looking into capacity building efforts or directly stated “what is in it for us?”

### Capacity building

Capacity building is a key aspect of our Centre’s mission [4]. This is achieved through a number of initiatives both university-wide and within our own Centre including supported Fellowships, hands-on engagement of students through the different aspects of research, and the attraction of PhD graduates to lift up our local research capacity to train future researchers [4].

Institutions cannot stand alone, and they cannot succeed nor progress if it were not for its people. We need quality people, who can be trained and will become engaged professionals contributing towards the advancement of society whilst addressing local needs. *Universidad Peruana Cayetano Heredia* is the leading institution in scientific production in Peru [13], with the highest output of scientific publications and with an overall normalised impact above the world’s average [13] as per international indicators [28]. *Universidad Peruana Cayetano Heredia* has trained health professionals in research for more than 50 years, and is constantly exploring and supporting alternative models of educational and research engagement [4, 29, 30].

#### Masters degree in epidemiological methods

Postgraduate training is essential for creating and sustaining networks and partnerships for future research opportunities. The Masters’ programme, organised by the *Universidad Peruana Cayetano Heredia* and the U.S. Naval Medical Research Unit No. 6 (NAMRU-6), provides extensive training in epidemiological methods training, coupled with hands-on experience in the preparation of a scientific publication as a mechanism for graduation. International researchers from the CRONICAS Centre of Excellence and many other groups in Peru rotate as faculty and mentors. Outstanding graduates are invited to join as teaching assistants and progress to becoming junior faculty, therefore expanding the base to train future generations.

#### CRONICAS research fellowships

Within Peru, our Centre of Excellence has pioneered the creation of a competitive Fellowship programme, the CRONICAS Scholarship, to support postgraduate training in the epidemiology of chronic diseases that includes full tuition support for a Masters in Epidemiological Research among other benefits.

The CRONICAS Fellowship is the first of its kind in Peru, and in only 5 years it has become extremely successful by multiple measurements. For example, we average 70 applicants per year for one Fellowship opportunity. The quality of our fellows is also very high: CRONICAS Fellows rank among the top Masters’ students in all 5 years since our inception. Nineteen CRONICAS Fellowships have been awarded to individuals with backgrounds in economics, medicine, nutrition, physical therapy, psychology, and statistics. To date, after completion of the Masters’ program, our Fellows have achieved different public health related positions, such as research project managers (4 fellows), chiefs of health departments’ units (2 fellows), and teaching assistants in a Masters program (4 fellows).

This speaks to our successful outreach, but also often leads us in new and exciting research directions that we would not have explored without the input from these Fellows from a range of scientific disciplines. Long after completing their Masters’ training, most of our former Fellows engage with our ongoing work: several graduates of the Masters’ programme joined CRONICAS as adjunct scientists, thus enhancing the mentorship they receive together with augmenting their exposure to research opportunities.

Most recently in early 2015, CRONICAS conducted an evaluation of its Fellowship programme using two different avenues. First, previous Fellows were tasked to review the experience and trajectories of the scholarship beneficiaries. They found that most of the beneficiaries were recently graduated from careers related to health with strong interest in research. However, most of them were not able to find a collaborative group and/or opportunities to develop their research careers. As one of the CRONICAS Fellows stated: “*It has been the best opportunity to enter into the world of scientific research with high international standards*”. Another Fellow described her experience at CRONICAS as: “*Finding the ideal working group to develop myself as a researcher*”.

In an open public consultation, using social media channels, we asked our external audience if the CRONICAS Scholarship should continue or not. We received many requests from our followers. We highlight two comments that summarize the demand for continuity CRONICAS Scholarship: “*This scholarship not only finances one of the best Masters’ programs in research in Peru but also offers the opportunity to be in an environment where research is a everyday activity, and you get apply what you learn on a daily basis, in addition to establish links with senior researchers and gaining access to sources of data from which you can develop new studies.*” Another one added, “*If one looks for research-oriented health institutions, there are very few of them. CRONICAS has set a good precedent to get more young people interested in research, its work should be continued and while this constitutes a major challenge … there are those willing to face them*.”

#### Hands-on engagement of students and young graduates

Another route for engagement is through learning-by-doing. In CRONICAS we welcome trainees to join our team, ideally for at least 1 year. This will enable them to learn about the various stages of research through active involvement. For example, some trainees have worked on hypothesis-driven secondary analysis of available data [31–33], while others support our ongoing projects or research initiatives, participating in project management, grant writing and writing-up scientific manuscripts. In this way, early-stage trainees receive mentoring and advice upon which they will develop their future interests and capacities. Under this scheme, trainees and visitors have been extremely productive, producing more than 70 first-authored publications under the mentorship of CRONICAS investigators [34].

CRONICAS Fellows and trainees are encouraged and welcomed to actively participate in proposing new research projects and to lead on grant applications. They receive critical advice from senior investigators and key input from specialists to develop a strong research idea. They also receive active support throughout the grant writing process. For example, CRONICAS trainees were awarded three seed grants from the National Heart, Lung and Blood Institute supplementary grants available through the Centres of Excellence network under a competitive application process [14]. Our trainees have led successful bids winning awards totalling USD $390,000 dollars to date.

#### Promoting and attracting high-calibre postgraduates

The repatriation scheme of PhD professionals promoted by Peruvian National Council for Science, Technology and Technological Innovation (CONCYTEC) was created in 2014 as a new opportunity and development pathway to enhance capacity building initiatives. Under this scheme, our Centre hosts a postdoctoral fellow trained in public health and medical anthropology. Additionally, one of our investigators has been awarded a Wellcome Trust Research Training Fellowship to pursue doctoral training in the UK, hence expanding the professional base of our Centre. Some of our trainees are further exploring other avenues for professional development thus expanding and nurturing the connection-base with other institutions. Two of our trainees are pursuing further career development opportunities in USA and UK. In the same vein, another one has been relocated to China for 2 months to work in specific research projects of mutual interest to both countries, therefore introducing a practical element of investing in sustaining the relationship between our research teams across the borders.

### Avoiding silo mentality

Our focus on partnership believes in the advantages of linking diverse areas of expertise as means to move forward in conducting research. Hence, it is vital that every single team member from the CRONICAS Centre, from junior to senior levels, together with international collaborators, step out of their silos and work together to maximise the returns of innovative research [4]. In this section we briefly present some of these interdisciplinary interactions.

#### Linking infectious disease and child health with non-communicable diseases

To research on non-communicable diseases does not mean to avoid infectious diseases or other conditions. We have investigated the impact of child growth in a setting where diarrhoea is highly prevalent on blood pressure and obesity [35, 36]. One of these studies showed that increased weight growth during infancy and early childhood was associated with decreased systolic blood pressure and central adiposity in adolescence, and the near 50 % rate of chronic under-nutrition baseline [37], may have impacted the observed associations between exposures and outcomes [38]. These findings may have not been found in other settings where the pattern of exposures and observed associations is completely different [39].

#### Hypoxia and human adaptation to high altitude

Moving beyond the physiological mechanisms underpinning high altitude hypoxia adaptive responses to include the role of other environmental factors is an area that has largely been neglected in the high altitude research agenda [40–42]. In Puno, a highland Peruvian area located at 3825 m above sea level, our international team of researchers showed that excessive erythrocytosis was strongly associated with having metabolic syndrome and being overweight, besides its association with hypoxemia and lower forced vital capacity [43]. Other high altitude research includes investigations of biomarkers linked to excessive erythrocytosis [44], use of point-of-care devices for screening of excessive erythrocytosis [45], and studies of the link between hypoxemia and cardiometabolic conditions [46].

#### Evolutionary biology

While diverse components of metabolism and body composition are implicated in the pathway to non-communicable diseases, the development of such variability can also be considered as adaptation to local ecological conditions. The ‘standard medical model’ pays inadequate attention to the variability in normal ranges of traits that occur across populations. An evolutionary perspective can help understand the local manifestation of broader pathways to disease, with the ultimate aim of identifying new potential targets for public health interventions. For example, the association between infant weight gain and later body composition is not uniform between high- and low-/middle-income populations [47], hence the twin challenges of reducing under-nutrition without exacerbating obesity risk must be resolved on a population-specific basis.

We conducted a study comparing growth and development between high altitude agropastoral communities (Vinchos and Santillana Districts of Ayacucho Region) and a low altitude urban community (Pampas de San Juan de Miraflores, Lima). We first showed that the shorter stature of the rural high altitude population was disproportionately in lower limb lengths, compared to hand/foot lengths, upper limb lengths or torso height [48]. This highlights how different components of growth are selectively sacrificed under ecological stress, while we also demonstrated that variability in arterial oxygen saturation is only one of the underlying mechanisms [49]. We further showed that short stature was associated with obesity risk in contrasting ways in these communities, such that stunting increased obesity risk in the rural high-altitude population, but decreased it in the urban low altitude population [50]. This suggests contrasting metabolic adaptations in association with growth, with implications for obesity prevention efforts. Finally, we also showed that Andean ancestry mediated the effect of altitude on the growth patterns [51].

In a separate study, we examined the impact on growth outcomes of a recent El Niño event, a climatic extreme to which Peru is regularly exposed. In the Tumbes region, we showed that being born at the time of the 1997 El Niño was associated with reduced height and lean mass in childhood, without any corresponding effect on fatness [52]. The same climate events also increase the prevalence of childhood diarrhoea [53]. Since height and lean mass are protective against diabetes, whereas body fat elevates risk, this stress may shape diabetes risk in adult life.

Collectively, these studies indicate that growth patterns are strongly shaped by both ancestry and ecological conditions, so that these factors shape the developmental pathway to chronic disease risk. An evolutionary approach aids interpret such data, and can help utilise the findings in public health efforts.

#### Individual-environment ecosystems and human vulnerability

One of our projects, combining team efforts between Brazil, Ecuador, Peru and USA, focuses on exploring the impact of anthropogenic land use/land cover and climate change on human and natural systems that affect the development of both infectious and non-communicable diseases in the Amazon. This initiative, supported by the Inter-American Institute for Global Change Research, brings together multidisciplinary experts to better understand socio-ecological vulnerability and resilience in rural tropical environments. The project is predicated on the idea that coupled land-climate change dynamics in the Amazon are primarily driven by human livelihood response, which refer to economic, environmental, and human health events that affect household risks. Managing this risk is a trade-off between the vulnerable state in which households exist and the resilience they have acquired to tolerate risk. This study will implement the same methodology and tools to collect data in Machadinho (Rondonia, Brazil), Madre de Dios (Peru), and the provinces of Succumbios and Orellana in the northern Ecuadorian Amazon to define a multi-dimensional metric of vulnerability and resilience that is sensitive to cultural, political, economic and demographic differences and directly applicable to current health threats.

### Knowledge translation, advocacy and policy development

Since its inception, our Centre has collaborated with the Ministry of Health’s National Health Strategy for Non-Communicable Diseases. In addition, our group has served different healthcare service providers and policy-oriented bodies including the Congress of Peru, Pan American Health Organization’s Advisory Committee for Health Research, Peruvian Diabetes Association, Peru’s National Institute of Health, Peru’s National Institute of Statistics and Informatics, Peru’s Social Security Health Service (EsSALUD), among other institutions. Whilst direct impact on policy cannot be documented, one of the most verifiable indicators is the recognition of our group by policymakers as an established research group, conducting meaningful research for the country’s needs including topics such as human resources for health [54], a health system analyses of barriers to hypertension and diabetes care [55], and the exploration of the burden of disability including caregivers [56]. Because of the strong ties we have developed with various of these organizations, our Centre tends to be invited or consulted for relevant policy forums, both at the national and regional levels.

Advocacy at the local level is closely tied to policy development. In the international arena, our Centre has advocated for region-specific diabetes forecasts [57], global responses to non-communicable diseases [58–60] including criticism towards the current predominance of north-only voices in the area [61], the need for flexibility and adaptation of global health curriculum [62], and the challenges of epidemiology in the Latin American region whilst actively advocating for South-South collaborations [63, 64]. In March 2015, our Centre hosted the second Emerging Leaders Think Thank Seminar, an ongoing programme of the World Heart Federation [65], thus contributing to advancing the prominence of Latin America in contemporary global health issues.

Our Centre is also required to actively participate in external activities and interactions, some of which serve as some form of knowledge translation or knowledge transfer and citizenship. This is particularly challenging in our Peruvian context, and at the international level, for two key reasons. One relates to our ability to be an active and meaningful contributor in every single arena. The interconnectedness between chronic non-communicable diseases with many other areas provides important opportunities to interact with many non-health actors at different levels, from transport to agriculture, to policy development and policy making, urban planning, and aid/development, to name a few. The other factor is the limited number of long-term interlocutors, particularly from the public sector, due to high turnover of key personnel within the system. Despite limited resources and time, we try to sustain participation in these different venues and with these different counterparts. The very same concept of building, nurturing and maintaining trustworthy relationships, as expressed in the case of our research collaborators, applies to our non-research partners, including policy makers and civil servants.

### Lessons learned: leadership, trust and time as assets towards ensuring sustainability

Another key component of any successful partnership is to learn from its experiences and in particular to learn from its failures, a key message constantly instilled by the group’s leaders. In this section we reflect and elaborate on key lessons learnt from the process of establishing a Centre of Excellence with a long-term vision. We place particular emphasis on how our Centre’s structure and activities fuse to contribute to ensuring its sustainability. Two key non-monetary assets, time and trust, are carefully scrutinised below.

#### Grant writing

The development of our Centre did not occur in isolation of funding support, which is crucial for its expansion and sustainability. While some funding opportunities are available to us without collaboration with a high-income country institution, in many cases it is either required or adds significant strength to our applications. As a result, as shown by the co-authorship base in this manuscript, we have gradually formed strong and trusted connections with researchers in high-income countries. Trust is a crucial component of these relationships and is a key to sustainability. Establishing and maintaining trust requires a significant investment of time and energy. This nurturing process is crucial as we learn to work together and to contribute to the advancement of mutual goals in a meaningful way.

Obtaining funding is one of the greatest challenges in academic research, and is particularly difficult in the context of transitioning economies such as Peru. Our Centre has little in-country support for research, and primarily relies on research funding from larger established funding bodies in Canada, UK and USA. While available international funding previously focused on infectious diseases research that were unique to low- and middle-income countries, recent initiatives recognise the value of global investigations into non-communicable diseases. Most of the funding available for research comes from international sources including public-private partnerships, though some opportunities that focus on training of local researchers are also required. The latter is a major challenge for Peru’s development, and has to be addressed locally.

#### Flexibility to expand into newer research areas

The core research group is entirely funded through research grants. Grant writing consumes a sizeable portion of our efforts, and it is crucial/essential to anticipate future periods of uncertainty. A practical limitation is that there is not always time to take away from ongoing research efforts to focus in new grant applications, which are often diverse and demands moving beyond our comfort zone. As progress in science today occurs largely at the boundaries of domains, working with multidisciplinary teams is a particular challenge, as part of our strategy for sustainability relies on expanding our portfolio of research. However, this is also positive because we constantly learn about new topics and approaches, pressing us to break down any signal of rooting into silo mentalities, as shown above in the diverse ongoing areas of partnership and collaborations. In the same vein, it hinders our capacity to specialise and reflect on the research findings as much as we would like. In practice, this scenario places us on a constant search for balance and equilibrium between risk aversion and spreading ourselves too thin. As discussed elsewhere, the Centre is not a risk-averse organisation: “it benefits from past experiences, including past mistakes, improves upon them, and challenges traditional research approaches. This ethos and environment is strategic to fostering innovation in research” [4].

#### Investing in students and early career professionals

Equally important is investing in younger generations, which includes securing and providing them with a meaningful and challenging space to make contributions. In this regard, part of our success in this short time can be attributed to capacity building support. As a research group, we strive to “do more” beyond answering our specific research questions, which requires that we invest our most valuable resource—time—to provide opportunities and mentorship to younger investigators to lead the preparation of manuscripts as well as grant proposals.

The flip side of the coin is that most of these ventures are not funded and require a major degree of commitment from the Centre’s investigators and the individuals in training. In addition, separate time and effort are also devoted to ensure adequate communication within the CRONICAS team, not only to monitor the progress of projects, but also to hear about the individual’s career development needs. Remaining actively sensitive to the career development paths of our trainees is not only about research methodology, but equally important, to develop and excel skills for cross-disciplinary and cross-national research, paired with skills to manage and develop their professional lives, as well as to ensuring that they remain engaged in their research.

#### Dynamic interactions

**Fig. 1:**
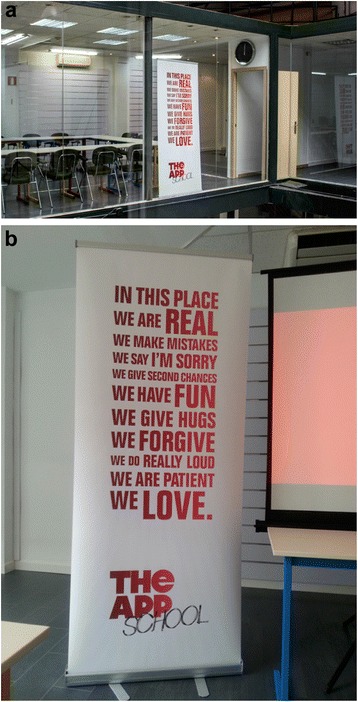
Fostering horizontally driven capacity building environments. Some features that promote an environment where mutual learning and horizontality is promoted (**a** and **b**). Source: The App School, http://theappschool.com/

Our Centre has put in place two strategies to ensure quality interactions and provides, on a day-to-day basis, a meaningful and challenging space to its members, from students to full-time investigators. First, we instituted a horizontal structure, in terms of relationships and office arrangements, which facilitates the exchange of ideas, discussions, project management tips, lessons learnt, and pitfalls to avoid in the future (see Fig. 1). Such structure enables fluid unplanned interactions, minimising hierarchies, between trainees, research project staff, and the Centre’s investigators. Second, we have a one-day per week open door policy for individuals or teams interested in the Centre’s activities to interact freely and openly with our staff. In so doing, our strategies affirm our Centre’s values of generosity, innovation, integrity and quality.

## Conclusions

The CRONICAS Centre of Excellence in Chronic Diseases at *Universidad Peruana Cayetano Heredia* is proud of its origins, thankful for its individual and institutional collaborators as well as its hosting environment, and appreciative of the initial funding from the National Heart, Lung and Blood Institute that enabled its creation [4, 5] as well as contributions from various other funding agencies. Within 5 years of its inception, the Centre has demonstrated that it is an established group, both in the Peruvian and international context.

More challenges will come, more adventures will follow, and the Centre will continue supporting the expansion of Peru’s critical mass available to conduct research, inform health policy and policy decision making, advance for global health, and continue to progress under its core values of generosity, innovation, integrity and quality.

The CRONICAS Centre’ experience described in this manuscript serves as an ongoing example of a Peruvian-led international actor in research whose experiences and trajectory ascertains important lessons for various partnerships whilst embracing multiple stakeholders. This experience fits nicely with the proposed definition of transboundary and intercultural research in partnership as a “continuous process of sound knowledge generation, building mutual trust, mutual learning and shared ownership” [26, 27]. Many other research groups and organisations located in the global South are well positioned to evaluate their current status of relationships, to make objective claims of true partnerships, and to contribute to the promotion of a culture of global solidarity where mutual goals, mutual gains, as well as mutual responsibilities are the norm. In so doing, we will all contribute to instil a new culture of expectations, roles and interactions among individuals and their teams, the true nature of partnerships.

The Centre’s presence in the Latin American region provides an additional anchor to maximize capacity building initiatives across countries. For example, the team’s dynamics and experiences described in this manuscript can be used as an example, out of many available, to inform about models of partnerships where costs of collaborations can be reduced and become more efficient in terms of investments and yield.

## Abbreviations

CONCYTEC, Consejo Nacional de Ciencia, Tecnología e Innovación Tecnológica; EsSalud, Seguro Social del Perú; KFPE, Swiss Commission for Research Partnerships with Developing Countries; NAMRU-6, U.S. Naval Medical Research Unit No. 6.
